# High-Survival Rate After Microinjection of Mouse Oocytes and Early Embryos With mRNA by Combining a Tip Pipette and Piezoelectric-Assisted Micromanipulator

**DOI:** 10.3389/fcell.2021.735971

**Published:** 2021-09-03

**Authors:** Lei-Ning Chen, Xiao-Yan Fan, Yi-Tong Liu, Shao-Qing Chen, Feng-Yun Xie, Li Zeng, Juan Wen, Jin Li, Jun-Yu Ma, Xiang-Hong Ou, Shi-Ming Luo, Lei Guo

**Affiliations:** ^1^Fertility Preservation Lab, Reproductive Medicine Center, Guangdong Second Provincial General Hospital, Guangzhou, China; ^2^Guangdong-Hong Kong Metabolism and Reproduction Joint Laboratory, Guangdong Second Provincial General Hospital, Guangzhou, China

**Keywords:** microinjection, cumulus–oocyte complex, GV oocyte, MII oocyte, zygote, two-cell embryo, piezo-assisted micromanipulator

## Abstract

Utilizing microinjection to introduce biological molecules such as DNA, mRNA, siRNA, and proteins into the cell is well established to study oocyte maturation and early embryo development *in vitro*. However, microinjection is an empirical technology. The cellular survival after microinjection is mainly dependent on the operator, and an experienced operator should be trained for a long time, from several months to years. Optimizing the microinjection to be highly efficient and quickly learned should be helpful for new operators and some newly established laboratories. Here, we combined the tip pipette and piezo-assisted micromanipulator to microinject the oocyte and early embryos at different stages of mouse. The results showed that the survival rate after microinjection was more than 85% for cumulus–oocyte complex, germinal vesicle oocyte, two-cell, and four-cell embryos, and close to 100% for MII oocyte and zygotes. The high-rate survival of microinjection can save many experimental samples. Thus, it should be helpful in studying some rare animal models such as aging and conditional gene knockout mice. Furthermore, our protocol is much easier to learn for new operators, who can usually master the method proficiently after several training times. Therefore, we would like to publicly share this experience, which will help some novices master microinjection skillfully and save many laboratory animals.

## Introduction

Delivery of desired molecules (especially nucleic acids) into living cells is a prerequisite of most biological research and treatments. According to the methods and reagents used, the delivery methods are classified into three categories: chemical, biological, and physical (Chou et al., 2011; Stewart et al., 2016, 2018). Mainly, the chemical methods include cationic lipid or cationic polymer-mediated delivery and calcium phosphate co-precipitation (Kim and Eberwine, 2010; Stewart et al., 2016). The biological method includes viral delivery (Samulski and Muzyczka, 2014). The physical methods include electroporation, laser-mediated transfection, biolistic particle delivery, and direct microinjection (Stewart et al., 2016, 2018). Benefiting from the development of biotechnology, many high-efficiency, and low-toxic transfection reagents such as liposomes, peptide-derived nanoparticles, and viral vectors have been rapidly developed in the last 20 years (Kim and Eberwine, 2010; Samulski and Muzyczka, 2014; Stewart et al., 2016). So the transfection that introduces nucleic acids such as mRNA and siRNA into cells is much easier than before. The mouse is one of the most important mammalian models to study reproduction because it has the characteristics of mammals identical to humans and a very high reproduction rate. However, since the mouse oocytes and early embryos are surrounded with a zona pellucida, and many transfection reagents are cytotoxic more or less, these chemical and biological transfection methods are not well suitable for them. Thereby, microinjection is still the most important method to deliver various biological materials into mouse oocytes and early embryos (Andras Nagy et al., 2003).

Microinjection is a technology that uses glass micropipettes to introduce nucleic acids, proteins, cytoplasm, organelles, microorganisms, and various other substances into living cells. It was invented by Dr. Marshall Barber, a bacteriologist at the University of Kansas School of Medicine. Originally, it was used to inoculate bacteria into living cells in 1911 (Barber, 1911). Since then, it has been developed for more than 100 years and used in many other cells such as nerve cells, *Xenopus* oocytes, and chicken embryos (Love et al., 1994; Wong et al., 2014; Aguero et al., 2018). In mice, Lin (1966) at the University of California San Francisco injected the bovine gamma globulin into the mouse pronucleus. Since then, as a commonly used technique, microinjection has been widely used in reproduction research in many laboratories. Generally speaking, the microinjection includes nuclear microinjection, cytoplasmic microinjection, and intracytoplasmic sperm injection (ICSI; Joris et al., 1998; Wall, 2001; Yuan et al., 2009; Rubino et al., 2016). The substances being introduced to a cell can be soluble or insoluble. In this study, we will, hereafter, refer to it as microinject mRNA, siRNA, proteins, and other liquid substances into the cytoplasm.

Unlike chemical and viral transfection, microinjection is a technique that relies more on experience (Joris et al., 1998; Andras Nagy et al., 2003; Jaffe et al., 2009; Kline, 2009; Stein and Schindler, 2011; Jin et al., 2016). The survival rate after microinjection largely depends on the operator and what the sample is injected. Typically, a skillful operator should be trained for a long time from several months to years. Therefore, it should be beneficial to establish a method to obtain a high survival rate for new operators after microinjection. Some monographs and papers have demonstrated the application and methods of microinjection in mouse reproduction (Andras Nagy et al., 2003; Jaffe et al., 2009; FitzHarris et al., 2018), but currently, there is no single protocol that could be used in all the samples from denuded oocyte, cumulus–oocyte complex (COC), and zygote to early embryos (Jaffe et al., 2009; Kline, 2009; Stein and Schindler, 2011). An easy-to-learn method is expected to be applicable to all the mouse oocytes and early embryos at different stages. Here, we optimized the microinjection through a combination of a tip pipette and piezo-assisted micromanipulator. The results demonstrated that the survival rate after microinjection could be about 85% for the COC, germinal vesicle oocyte, two-cell and four-cell embryos, and nearly 100% for MII oocyte and zygotes.

## Materials and Methods

This study aims to improve the method of microinjecting liquid material into mouse oocytes and early embryos. The readers may have the basic knowledge of mouse reproduction. If anyone is not familiar with mouse reproduction, we recommend a monograph (Manipulation of Mouse Embryos: Laboratory Manual) edited by Andras Nagy et al. (2003).

### Mouse Oocyte and Embryo Preparation

All animal experiments in this study were carried out under the guidelines for animal experiment standards in the Guangdong Second Provincial General Hospital. 8-week-old ICR mice were purchased from Beijing HFK Biotechnology Co., Ltd. and raised during the 12/12-h light/dark period. Pregnant mare serum gonadotropin (PMSG) and human chorionic gonadotrophin (hCG) were purchased from Ningbo Animal Hormone Factory, Ningbo, China. Female mice were intraperitoneally injected with 10 IU of PMSG and 10 IU of hCG over a 48-h interval for superovulation. The mice were sacrificed by cervical dislocation 44–48 h after PMSG injection to obtain COC, or 13 and 16 h after hCG injection to obtain MII oocytes and fertilized eggs, respectively. The oocytes and fertilized eggs were collected with M2 medium, and then cultured in M16, or KSOM droplets covered with mineral oil (M5310, Sigma, St. Louis, MO, United States), respectively, and developed at 37∘C and 5% CO_2_.

### Plasmid Construction and mRNA Preparation

GFP-labeled actin plasmid was a gift from Addgene (mEmerald-Actin-C-18, #53978). To construct a GFP plasmid that can be transcribed *in vitro*, the actin sequence was removed, and a T7 promotor (TAATACGACTCACTATAG) was inserted with the cloning and recombination kit (ClonExpress II One Step Cloning Kit, C112, Vazyme Biotech Co., Ltd., China). The GFP mRNA was then obtained with an RNA transcription kit [HiScribeTM T7 ARCA mRNA Kit (with tailing), E2065S, New England Biolabs, United States] and divided as 0.8 μl each tube and stored at −80∘C until use.

### Microinjection Needle Preparation

**FIGURE 1:**
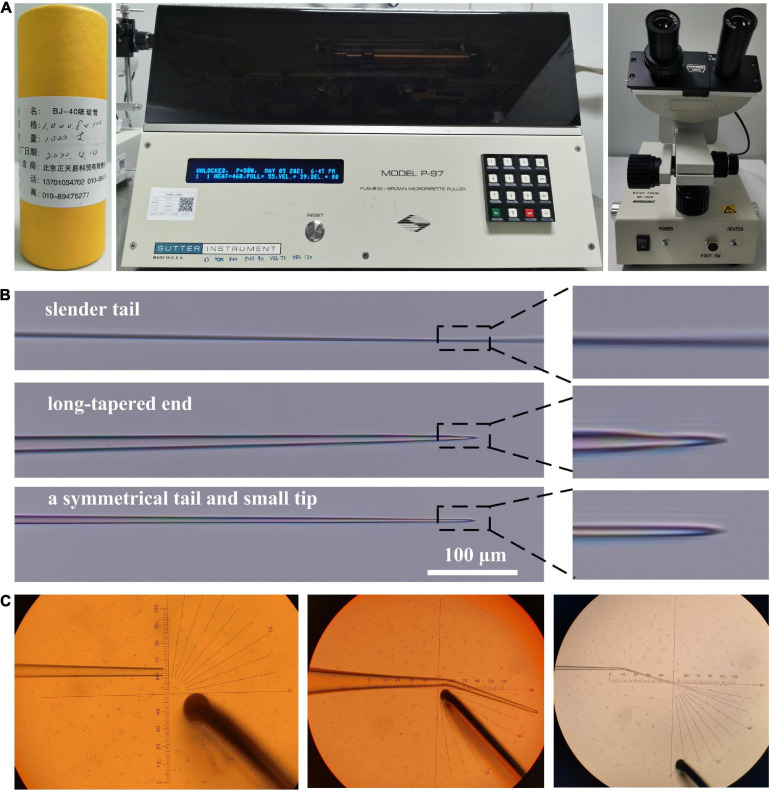
Making holding and microinjection pipettes. **(A)** The capillary glass tube, the micropipette puller, and the microforging were used to make the holding and microinjection needles in this study. **(B)** Three types of the needle are produced from the micropipette puller. The needles with a symmetrical tail and small tip are ideal to make microinject pipettes. **(C)** The cutting diameter should be about 50 μm, which is good for making a holding pipette (the objective lens is ×10). At a position about 2.5 mm away from the end of the pipette, the holding and microinjection needle will be bent for 15–20° (the objective lens is ×5).

Capillary glass tubes (outer diameter 1 mm, inner diameter 0.8 mm, and length 10 cm), micropipette puller (P-97, Sutter Instrument, CA, United States), and microforge (MF-900, Narishige, Tokyo, Japan) were used in this study to prepare the microinjection needles (Figure 1A). The capillary glass tube was fixed on the micropipette puller and pulled out as two needles per the instructions of the manufacturer, and then processed as a holding and microinjection pipette. In our method, the microinjection pipette is the most important factor in determining the result of the microinjection. As shown in Figure 1B, according to the parameters of the micropipette puller, three types of needles can be produced: one with a slender tail, the other with a long-tapered end, and the last with a symmetrical tail and small tip, which were subsequently used in our method to make the microinjection pipette. After the two needles were made, one of them was cut with a grinding wheel to make a holding pipette. The cut diameter should be 30∼70 μm (Figure 1C), and the other one should be lightly hit once or twice with the glass ball of the microforge to make a microinjection pipette. It should be noted that the incision of the microinjection pipette may be too small to be seen under the microforge. Finally, the microforge was used to shrink the incision of the holding pipette, and it and the microinjection pipette were bent to a certain extent. Generally, the bending position and angle will not affect the microinjection, while we wanted to bend it to about 15–20° at a position 2.5 mm away from the end of the pipette (Figure 1C).

### Microinjection Sample and Operation Dish Preparation

**FIGURE 2:**
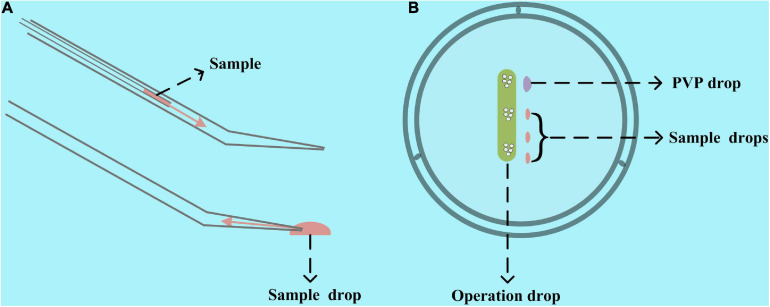
Schematic diagram of preparing the operation dish. **(A)** Two ways to add samples to the microinjection pipette. One is from the tail with another pipette injecting the sample, and the other is to use the microinjection pipette itself to aspirate the sample from its injection tip. The second one was used in our study. **(B)** An operation and PVP drop and one or several sample drops are included in the operation dish. A lid rather than a 35- or 60-mm Petri dish itself would be a better container for making the operation dish.

There are two ways to add mRNA, siRNA, and other microinjection samples into the microinjection pipette. One is to use another pipette to add the samples from the back of the microinjection pipette, and the other is to use the microinjection pipette itself to aspirate the sample from its injection tip (Figure 2A). Compared with the first one, the second one has two advantages. It requires fewer microinjection samples and does not need to change the microinjection pipette for multisample microinjection. Thereby, we used the second one in our study and included the microinjection samples in the operation dish. Before preparing the operation dish, all the liquid, including the M2 operation liquid, 7% polyvinylpyrrolidone solution (ART-4005, SAGE IVF Inc., CT, United States), and microinjection samples were centrifuged at 10,000 × *g* for 1 min to remove the impurities. Then, as shown in Figure 2B, 1–2 μl of PVP drop for cleaning the microinjection pipette, 0.6–2 μl of microinjection samples, and about 30 μl of M2 operation liquid were included in the operation dish covered with mineral oil. It is worth noting that we would like to use the lid instead of the 35- or 60-mm Petri dish itself and prepare the M2 operation droplet as a long strip instead of a spherical drop in our method. These operations will enable our microinjection pipette to have a longer range of motion and make the oocytes and embryos in the operating drop remain stationary when moving the operating dish. Therefore, we can simultaneously microinject multiple types of oocytes and embryos in one operation drop without mixing them.

### Installation of Micropipette

**FIGURE 3:**
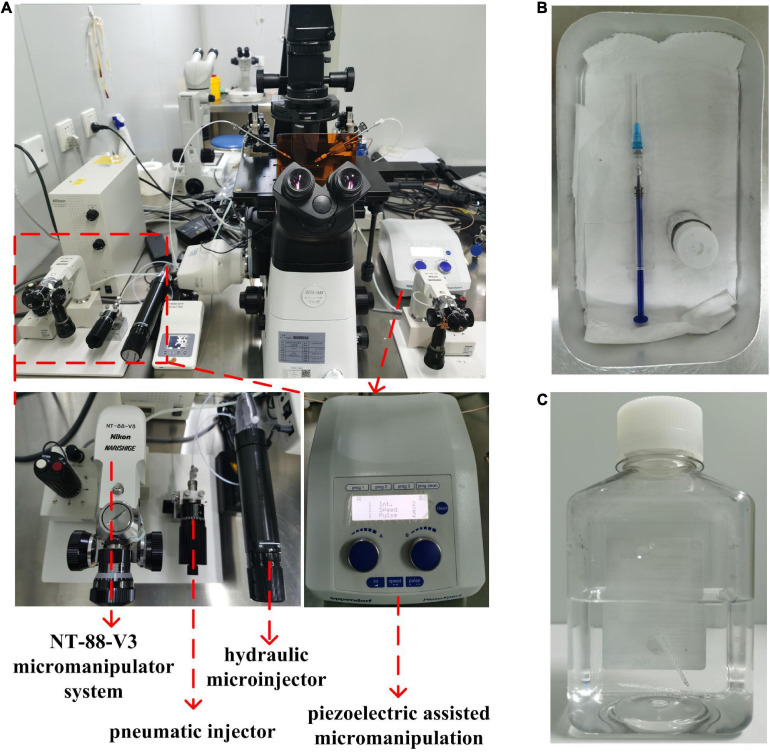
The microinjection equipment used in this study. **(A)** An inverted microscope, a micromanipulator system (Narishige NT-88-V3, Japan), a pneumatic injector (IM-9C, Narishige, Japan), an oil hydraulic manual microinjector (CellTram Oil, Eppendorf AG, Hamburg, Germany), and a piezoelectric-assisted micromanipulation (PiezoXpert, Eppendorf AG, Hamburg, Germany) were the microinjection equipment used in this study. **(B)** The mercury covered with water is stored in a glass bottle and added to the microinjection pipette in an aluminum dinner bucket with a syringe. **(C)** The microinjection needles were recycled in a bottle filled with water.

As shown in Figure 3A, an inverted fluorescence microscope (ECLIPSE Ti2-U, Nikon Corporation, Japan), equipped with Nikon Advanced Modulation Contrast and Differential Interference Contrast technology, a glass thermal plate (Tokai Hit, Japan), and a micromanipulator System (Narishige NT-88-V3, Japan), was used as the micromanipulation platform in our study. The microinjection pumps used to install the holding needle and microinjection needle are a pneumatic injector (IM-9C, Narishige, Japan) and oil hydraulic manual microinjector (CellTram Oil, Eppendorf AG, Hamburg, Germany), respectively. In addition, in our study, piezoelectric-assisted micromanipulation (PiezoXpert, Eppendorf AG, Hamburg, Germany) was used to provide the impulse to penetrate the zona pellucida and cell membranes. To stabilize the microinjection needle tip, we added a little mercury to it with a syringe, as shown in Figure 3B. Then, we used a lighter to bake the ends of the holder and the microinjection needles to reduce damage to the rubber ring of the microinjector, and then installed them on the pneumatic syringe and hydraulic manual microinjector for microinjection. Here, according to our experience, the volume of mercury and the roasting degree of the needle will not affect the microinjection. Finally, the holder and microinjection needle were moved to the focal plane of the inverted microscope with the micromanipulator system, and the microinjector was used to squeeze out the air in the microinjection needle (Supplementary Video 1). What needs special attention here is that mercury is toxic and volatile, so it should be handled with care and kept by someone. In our laboratory, mercury is covered with water and stored in glass bottles. Mercury-containing glass bottles and syringes are stored in an aluminum dinner bucket, and mercury-containing microinjection needles will be recycled in water-covered bottles after use (Figure 3C). In addition, the process of adding mercury to the microinjection needle should be performed on the dinner bucket, so that if some mercury leaks out, it will be confined in the dinner bucket and easy to be cleaned.

### Microinjection

**FIGURE 4:**
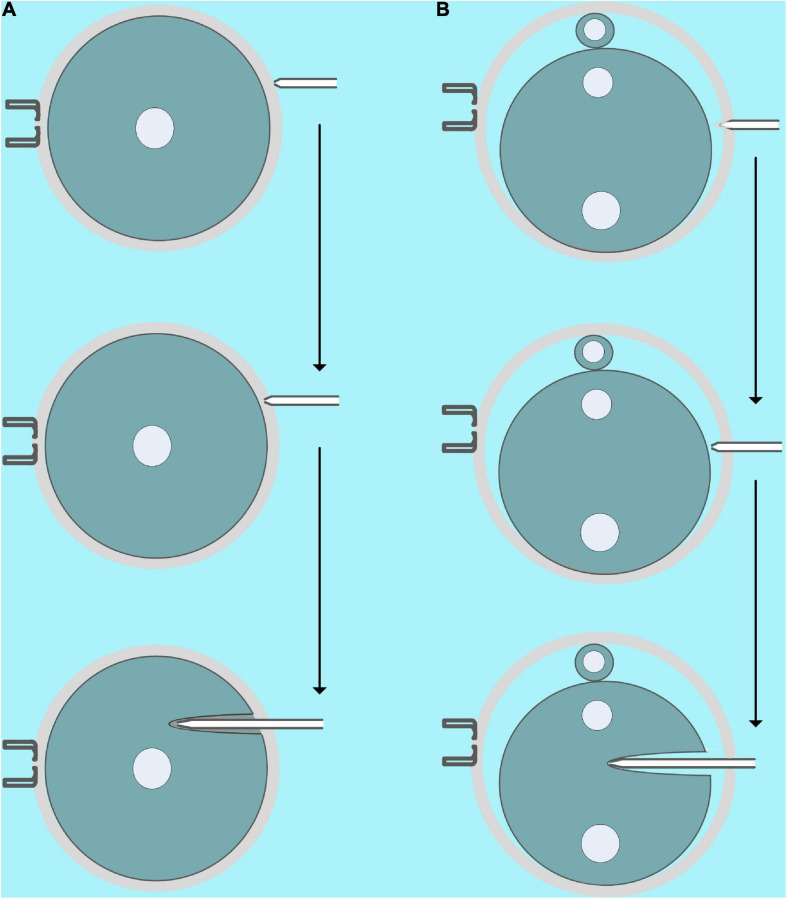
Schematic diagram of microinjection. **(A)** Microinjection diagram of GV oocytes [microinjection of cumulus–oocyte complex (COC) is similar]. First, the microinjection pipette should be close to, instead of pressing, the zona pellucida, and then a small piezoelectric pulse was used to incompletely penetrate the zona pellucida. Finally, the microinjection needle with the incomplete zona pellucida and the cell membrane is inserted into 1/3 to 2/3 of the oocyte, and then the oil pressure microinjector is turned into a vomiting state, and a minimum amount of piezoelectric pulse is given at the same time. **(B)** Microinjection diagram of fertilized eggs (microinjection of MII oocytes, two- and four-cell stage embryos are similar). First, the microinjection pipette presses the zona pellucida, and then an intermediate piezoelectric pulse is used to completely penetrate the zona pellucida. Finally, the microinjection needle is inserted into 1/3 to 2/3 of the cell, and then the oil pressure microinjector enters the vomiting state, and the smallest amount of piezoelectric pulse is given at the same time.

In this study, we microinjected COC, GV, and MII oocytes, zygotes, two-, and four-cell embryos with mRNA. Except for some details, the microinjection process for a different objective is similar. First, the microinjection needle is cleaned several times with the microinjector in the PVP drop, and then some PVP is aspirate drawn to separate the mercury and mRNA samples. Finally, the mRNA samples are aspirate drawn to the needle (Supplementary Video 2). It is worth noting that a bit of air in the microinjection needle will not affect the microinjection, so mercury should not be squeezed into the PVP droplets because it is toxic. In addition, the volume of mRNA in the microinjection needle is an important factor in reducing the intensity of piezoelectric-assisted micromanipulation, so we can use it to control the intensity of piezoelectric-assisted micromanipulation in actual operations. Generally, we can aspirate draw more mRNA for microinjection of COC and GV oocytes because their cell membranes are fragile, while microinjection of MII oocytes and early fertilized eggs need to aspirate draw less mRNA because their cell membrane is more flexible. As shown in Figure 4A and Supplementary Videos 3, 4, for COC and GV oocytes, we first moved the microinjection needle close to the zona pellucida, and then a small piezoelectric pulse was used to penetrate it incompletely. Subsequently, we inserted the microinjection needle with the incomplete zona pellucida and cell membrane into the 1/3 to 2/3 of the oocyte, and then the oil pressure microinjector was turned into a state of vomiting and, at the same time, gave the oocyte a minimum amount of Piezo pulse. When we saw that the injection was completed, we quickly pulled out the microinjection needle. For MII oocytes and embryos of different stages, due to the clear perivitelline space, we used higher piezoelectric pulses to completely penetrate the zona pellucida, and then the microinjection was completed similar to GV oocytes (Figure 4B and Supplementary Videos 5–8).

### Fluorescence Imaging and Analysis

To confirm the success of each microinjection, we injected a GFP-encoding mRNA instead of other samples (such as siRNA) into the target oocytes and embryos, and subsequently, the GFP fluorescence was imaged with a high-speed spinning disk confocal microscopy (Andor Dragonfly 200, Andor Technology, Belfast, United Kingdom), and its fluorescence intensity was obtained by ImageJ.

### Data Analysis

Each experiment was repeated more than three times in this study, with more than 10 cells in each sample. The statistical results were analyzed by Origin2019 and expressed as mean ± SEM.

## Results and Discussion

Microinjection is one of the most powerful technologies to study reproduction and early embryo development (Wall, 2001; Andras Nagy et al., 2003; FitzHarris et al., 2018). By utilizing it, people learned a lot of knowledge about oocyte maturation and embryo development. However, microinjection is much more empirical; its success depends mainly on the operator and the injected cells. Oocyte maturation and early embryo development are highly programmed, during which the oocytes and embryos quickly complete various biological events such as meiosis maturation, fertilization, and embryo activation. The cell membranes of oocytes and embryos at different stages are very different, which involves diverse microinjection methods (Jaffe et al., 2009; Kline, 2009; Stein and Schindler, 2011; FitzHarris et al., 2018).

For mouse oocytes, the GV stage is very fragile and can be directly microinjected with a tip needle, while in the MII stage, their membrane is very soft, so an electric-assisted microinjection or piezoelectric-assisted micromanipulator is useful (Yoshida and Perry, 2007; FitzHarris et al., 2018). Piezoelectric-assisted micromanipulators are widely used in various micromanipulations such as ICSI and enucleation/nuclear transfer to penetrate the zona pellucida and membranes with impulse (Yoshida and Perry, 2007). A blunt needle is considered necessary for these purposes because it has the best mechanical force transmission (Hirabayash et al., 2002). Here, we infer that a tip needle produced from a capillary glass tube should be similar to a blunt needle, so we can use it to penetrate the zona pellucida and cell membrane with a piezoelectric-assisted micromanipulator, and considering that the tip needle may damage the cell membrane very slightly, the survival rate after microinjection will be improved.

According to the elasticity of cell membranes, we divide mouse oocytes and embryos into three types. GV oocytes and COC are the first types. These cells are very fragile. It is easy to penetrate their membranes with a tip or even a blunt needle. However, the blunt needle will form a larger hole and rupture the cells, which cannot be healed automatically. Zygotes, two-, and four-cell embryos are the second types, the flexibility of which cell membrane is moderate. So both the tip needle and blunt needle combined with a piezoelectric-assisted micromanipulator can be used for their microinjection. MII oocytes are the third type. These cells are so soft that even a sharp needle cannot easily penetrate the cell membrane. Therefore, piezoelectric-assisted micromanipulators are usually used in their microinjection. Here, we used the optimized method to microinject all the three types of cells. As mentioned above, microinjection is a technique that relies heavily on experience so that the survival rate may vary significantly between different laboratories and operators, even with the same method. Therefore, we only showed the results obtained from our optimized method rather than comparing it with other microinjection methods. The results in Table 1 and Supplementary Videos 3–8 show that the survival rate after microinjection of GV oocytes, COC, two-, and four-cell embryos is more than 85%, and that of MII oocytes and fertilized eggs is nearly 100%. Furthermore, the blastocyst development rate of fertilized eggs, two- and four-cell embryos after microinjection was also examined. The results are shown in Table 2, which are nearly 100%.

**TABLE 1:** Statistics of microinjection results.

**Sample**	**Replication no.**	**Number of injections**	**Number of survival**	**Survival rate (%)**	**Average survival rate (%)**
GV oocytes	1	30	30	100	90.4
	2	41	35	85.36	
	3	21	18	85.71	
COC	1	32	28	87.5	86.8
	2	32	29	90.63	
	3	28	23	82.14	
MII oocytes	1	27	27	100	98.6
	2	30	30	100	
	3	24	23	95.83	
Zygotes	1	30	29	96.67	98.9
	2	15	15	100	
	3	21	21	100	
2-Cell embryos	1	25	25	100	88.53
	2	24	22	91.67	
	3	23	17	73.91	
4-Cell embryos	1	25	23	92	89.72
	2	15	13	86.67	
	3	21	19	90.48	

**TABLE 2:** Statistics of blastocyst development results.

**Sample**	**Replication no.**	**Number of survival**	**Number of blastula**	**Blastocyst development rate (%)**	**Average blastocyst development rate (%)**
Zygotes	1	29	29	100	98.41
	2	15	15	100	
	3	21	20	95.23	
2-Cell embryos	1	25	22	88	94.45
	2	22	21	95.45	
	3	17	17	100	
4-Cell embryos	1	23	21	91.3	97.1
	2	13	13	100	
	3	19	19	100	

**FIGURE 5:**
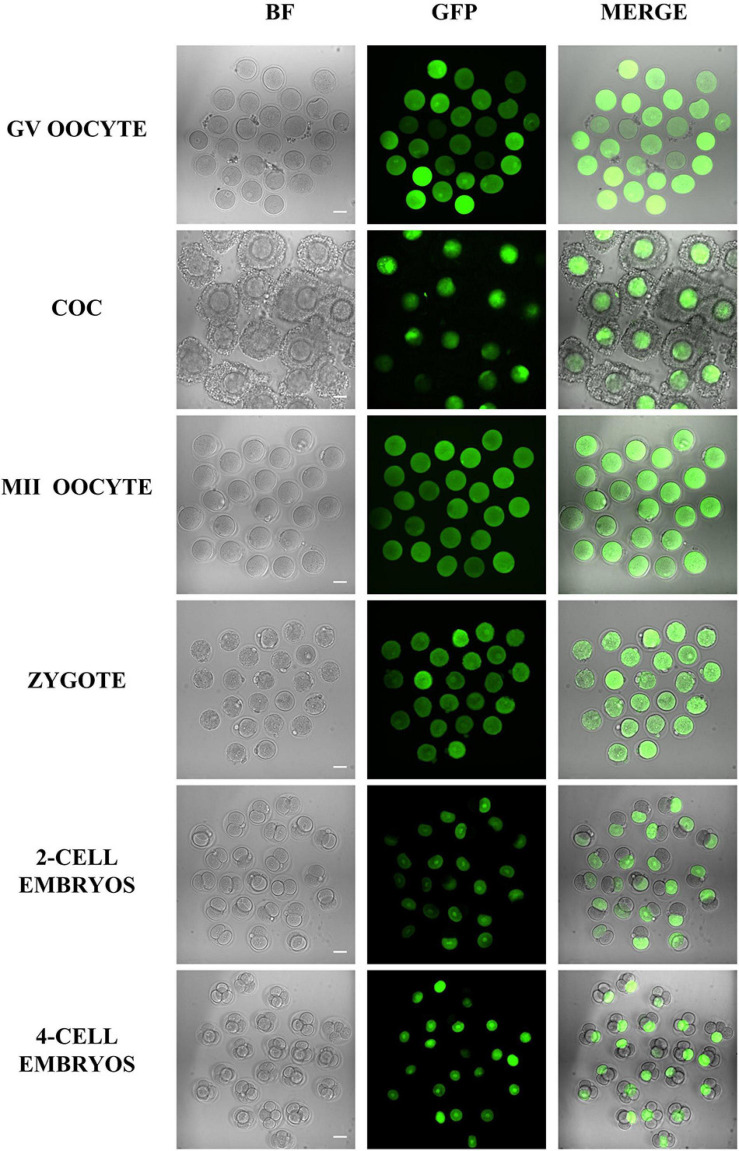
Representative fluorescence images of oocytes and embryos after microinjection. Different oocytes and embryos were microinjected with GFP mRNA and imaged 5 h later. The results showed that all samples were successfully injected, and except for some COC and four-cell embryos that were not on the focal plane, most of the samples had similar GFP fluorescence. Bar = 50 μm.

Besides survival and developmental rate, the microinjection volume is another factor that should be considered for microinjection. Here, we injected the mRNA encoding GFP into the oocytes and embryos, so then we could easily evaluate the microinjection effects by the fluorescent signals. The results in Figure 5 indicate that the GFP intensity between different cells is highly similar, which means that the volume of microinjected mRNA is similar and stable.

Unlike other samples such as cultured cells and tissues, mammalian oocytes, and embryos are relatively scarce. Therefore, the shortage of samples is one of the most important factors limiting the research progress in reproduction and early embryo development, especially for certain mouse models, such as aging, metabolic diseases, and some genome-edited mice with fewer oocytes. Thereby, improving the survival rate after microinjection is very useful for these studies. Here, the microinjection method in this study can have a high survival rate of over 85% for all tested samples, which will enable better utilization of rare oocytes and embryos. In addition, our method is applicable to all samples from fragile GV oocytes to soft MII oocytes, which means that people can use it for microinjection of different samples at the same time and significantly improve the experimental efficiency. In our method, the shape of the micropipette and the use of piezoelectric-assisted micromanipulators are the two most important factors in determining the microinjection results. Since they are relatively stable and more accessible to control, the method is less dependent on experience and more accessible to be mastered by the novice. Generally speaking, in our laboratory, the new users usually master it proficiently after several times of training. Therefore, this optimized microinjection method is very friendly to new users.

## Conclusion

In summary, this study demonstrates that using a particular tip micropipette combined with a piezoelectric-assisted micromanipulator can significantly improve the survival rate and efficiency of mouse oocyte and embryo microinjection. Microinjection as a prevalent technique is now widely used in reproductive research. The method described here will be helpful to some new users and newly established laboratories, which can save the experimental animals and improve experimental efficiency.

## Data Availability Statement

The original contributions presented in the study are included in the article/Supplementary Material, further inquiries can be directed to the corresponding author/s.

## Ethics Statement

The animal study was reviewed and approved by All animal experiments in this study were carried out under the guidelines for animal experiment standards in the Guangdong Second Provincial General Hospital.

## Author Contributions

L-NC, X-YF, S-ML, and LG conceived and designed the experiments. X-YF and others conducted experiments. X-YF and S-ML analyzed the data. S-ML wrote the first draft of the manuscript and the others revised the manuscript. All authors contributed to the article and approved the submitted version.

## Conflict of Interest

The authors declare that the research was conducted in the absence of any commercial or financial relationships that could be construed as a potential conflict of interest.

## Publisher’s Note

All claims expressed in this article are solely those of the authors and do not necessarily represent those of their affiliated organizations, or those of the publisher, the editors and the reviewers. Any product that may be evaluated in this article, or claim that may be made by its manufacturer, is not guaranteed or endorsed by the publisher.

## Supplementary Material

Supplementary Video 1 Use the microinjector to squeeze out the air from the microinjection needle. Because the tip of the needle is small, the air is discharged slowly from the inside at first, and then discharged at an extremely fast speed. The mercury will not be discharged from the tip of the needle.

Supplementary Video 2 Aspirating the mRNA sample. The microinjection needle is cleaned several times with the microinjector in the PVP drop, and then carrying some PVP to the mRNA sample drop to aspirate mRNA.

Supplementary Video 3 Microinjection of GV oocytes. Due to the lack of the perivitelline space and the fragile cell membrane, small piezoelectric pulses should be used to incompletely penetrate the zona pellucida so as not to damage the oocytes. Bar = 73.5 μm.

Supplementary Video 4 Microinjection of COC. It is similar to the microinjection of GV oocytes. However, due to changes in the number of cumulus cells, the focal plane needs to be adjusted continuously. Bar = 73.5 μm.

Supplementary Video 5 Microinjection of MII oocytes. MII oocytes are the most flexible samples. Bar = 73.5 μm.

Supplementary Video 6 Microinjection of fertilized eggs. Bar = 73.5 μm.

Supplementary Video 7 Microinjection of 2-cell embryos. Adjusting the two blastomeres to the same focal plane is more conducive for microinjection. Bar = 73.5 μm.

Supplementary Video 8 Microinjection of 4-cell embryos. Bar = 73.5 μm.
